# Substance Use (Alcohol, Areca Nut and Cigarette) Is Associated with Poor Prognosis of Esophageal Squamous Cell Carcinoma

**DOI:** 10.1371/journal.pone.0055834

**Published:** 2013-02-07

**Authors:** I-Chen Wu, Chun-Chieh Wu, Chien-Yu Lu, Wen-Hung Hsu, Meng-Chieh Wu, Jui-Ying Lee, Shah-Hwa Chou, Jang-Ming Lee, Yi-Ping Chou, Deng-Chyang Wu, Ming-Tsang Wu

**Affiliations:** 1 Division of Gastroenterology, Department of Internal Medicine, Kaohsiung Medical University Hospital, Kaohsiung, Taiwan; 2 Department of Medicine, Faculty of Medicine, College of Medicine, Kaohsiung Medical University, Kaohsiung, Taiwan; 3 Department of Pathology, Kaohsiung Medical University Hospital, Kaohsiung, Taiwan; 4 Division of Internal Medicine, Kaohsiung Municipal Hsiao-Kang Hospital, Kaohsiung, Taiwan; 5 Division of Chest Surgery, Department of Surgery, Kaohsiung Medical University Hospital, Kaohsiung, Taiwan; 6 Department of Surgery, National Taiwan University Hospital, Taipei, Taiwan; 7 Department of Surgery, Kaohsiung Veterans General Hospital, Kaohsiung, Taiwan; 8 Department of Family Medicine, Kaohsiung Medical University Hospital, Kaohsiung, Taiwan; 9 Department of Public Health, Kaohsiung Medical University, Kaohsiung, Taiwan; 10 Center of Environmental and Occupational Medicine, Kaohsiung Municipal Hsiao-Kang Hospital, Kaohsiung, Taiwan; Vanderbilt University Medical Center, United States of America

## Abstract

**Background:**

Few studies have reported the association between lifestyle factors and prognosis of esophageal squamous cell carcinoma (ESCC) and among these, the effects of habitual areca nut chewing have never been examined.

**Methodology/Principal Findings:**

Data from 718 pathology-proven ESCC patients recruited in a multicenter hospital-based case-control study between 2000 and 2008 in Taiwan were analyzed. Clinical and lifestyle information were obtained by chart review and questionnaire survey. Death was confirmed using the National Death Index. The mean age at diagnosis was 59.8 years and 506 (70.5%) patients presented with stage III or IV diseases. The overall 1- and 5-year survival rates were 41.8% and 9.75% respectively. In addition to clinical stage, habitual alcohol drinking was found to be the strongest predictor for ESCC survival, followed by areca chewing and smoking. Compared with non-users, patients who regularly used all three substances (alcohol, areca nut, and cigarette) had 1.52 times the risk of early death (adjusted hazard ratio = 1.52, 95% CI = 1.02–2.27, *p = *0.04). In addition, the more the number of substances used, the worse the prognosis of ESCC (adjusted *p* for trend = 0.01).

**Conclusions/Significance:**

Our study found that indulgence in more substances is a significant predictor of ESCC survival. Further mechanistic studies are necessary to elucidate how these substances lead to an adverse outcome.

## Introduction

Esophageal cancer is the 6th leading cause of cancer death worldwide, estimated to be responsible for 406,800 deaths in 2008 [1]. Squamous cell carcinoma and adenocarcinoma are the two major histological types of esophageal cancer. Contrary to the changing prevalence in Western countries, >95% of esophageal cancer in Taiwan is squamous cell carcinoma and the incidence has doubled during the past 10 years among Taiwanese men [2]. Despite the advances in diagnosis and treatment, most patients present with late stage diseases and suffer from tumor recurrence and metastasis. The overall 5-year survival rate for esophageal cancer is less than 15%. However, with complete tumor resection, the 5-year survival rate exceeds 95 percent for stage 0 disease, and is 50 to 80 percent for stage I disease [3].

Alcohol and tobacco use are well-recognized risk factors of esophageal squamous cell carcinoma (ESCC) worldwide [4], [5]. Recently, cumulative evidence indicates that areca nut chewing leads to ESCC and it has been classified as a group I carcinogen for this disease [6], [7]. Combination uses of alcohol, tobacco and areca nut have multiplicative effect on the development of ESCC. Moreover, our recent report linked them to the early onset of ESCC [8]. Such information suggests that lifestyle factors, especially substance use (alcohol, areca nut and cigarette), should be considered when determining the starting age of future ESCC surveillance. In contrast, few studies have explored the influence of lifestyle factors on esophageal cancer survival [9]–[12]. Two early studies from Sweden and Japan showed cigarette smoking may play a pivotal role in the prognosis of ESCC [9], [10]. However, a recent report including 301 Australian ESCC patients indicated that heavy consumption of alcohol, but not cigarettes, was associated with worse survival of ESCC [11]. Because of the conflicting findings plus the influence of areca nut chewing that has not yet been examined, we analyzed the effect of substance use on ESCC survival in Taiwan.

## Materials and Methods

### Study Subjects

A multicenter cancer patient recruitment for a molecular epidemiologic investigation was prospectively conducted in three medical centers, including National Taiwan University Hospital (NTUH), Kaohsiung Medical University Hospital (KMUH) and Kaohsiung Veterans General Hospital (KVGH) in Taiwan since 2000. The detailed study design has been described previously [8]. In brief, the study subjects were newly diagnosed ESCC patients mainly from the Department of Chest Surgery of NTUH and KGVH and from both Departments of Chest Surgery and Gastroenterology of KMUH. According to our previous studies [7], [13], the participation rate was 71.5% in NTUH and ∼95% in both KMUH and KVGH. In order to have enough follow-up periods, only those diagnosed before October, 2008 were analyzed in this study. Patients who died within one month after treatment were excluded because the short survival was possibly related to treatment complications rather than personal habits.

### Tumor Staging, Treatment Modality and Patient Follow-up

Clinical and pathological features were reviewed and evaluated by independent pathologists according to the TNM staging system of the American Joint Committee on Cancer (AJCC) [14]. The treatment decisions were based mainly on the initial TNM staging and the presence of organ insufficiency. The details have been described in our previous study [15]. In brief, patients with resectable disease and normal organ function, radical esophagectomy with lymph node dissection was strongly recommended. Adjuvant chemoradiation or concurrent chemoradiation therapy (CCRT) were performed after operation for those with advanced ESCC (T3-4 or positive lymph node metastasis). For those with clearly unresectable disease (T4b or stage IV) or very high surgical risk, definitive chemoradiation is indicated. The survival period was the interval between the date of pathological proof of ESCC and the date of death or the end of follow-up (December 31, 2008). For those lost to follow-up, we confirmed their status by linking their identification to the National Death Index, which contains records of all deaths in Taiwan, at the endpoint of December 31, 2008. All-cause mortality was used as the primary endpoint for follow-up.

### Questionnaire

A standardized questionnaire was used to collect comprehensive information of demographic characteristics and substance use via a personal interview with participants within one week of cancer diagnosis. Alcohol drinkers, tobacco smokers and areca nut chewers were defined, respectively, as subjects who had consumed any alcoholic beverage ≥1 times per week, those who had smoked ≥10 cigarettes per week, and those who had chewed ≥1 areca-quid per day for at least 6 months. The age at which a substance use started, type of substances, daily consumption amount and duration of such use, were documented for each participant [13]. The accuracy of information on substance use obtained from questionnaires has been validated by different experiments and described in detail in our previous work [8]. Other information such as education level (<high school, high school, and >high school) was also collected. The ethics review board of Kaohsiung Medical University Hospital reviewed and approved this investigation. Written consents were obtained from all participants.

### Statistic Analysis

Means ± standard deviation (SD) and medians (interquartile range, IQR) of the survival period (months) were compared according to the category of demographic and clinical variables. Differences of means between two or ≥ three groups were tested using the independent *t-*test or ANOVA statistics.

The main interest of this study was to examine the effect of substance use on the prognosis of ESCC. Since there is a high correlation between cigarettes and alcohol (kappa = 0.51, *p = *0.003), which might introduce co-linearity, we plotted the Kaplan–Meier survival curve according to the number of substances used. Log rank tests were used to assess any heterogeneity of the number of substances used in survival curves. Hazard ratios (HR) and 95% confidence intervals (95% CI) were obtained from Cox proportional hazards regression analysis before and after adjusting for other covariates, including age, sex, AJCC stage (I, II or III, IV) and education level (< high school, high school or >high school). To test for the linear trend, we modeled the category as a continuous variable. All tests were performed by SAS 9.3 statistical software; two-sided *p* value <0.05 was considered as significant.

## Results

In total, 718 ESCC patients (674 men and 44 women) were analyzed. The mean age (±SD) at cancer presentation of was 59.8 (±11.6) years. A total of 624 patients (89.9%) died during follow-up. The overall 1- and 5-year survival rates were 41.8% and 9.75% respectively. The survival period was comparable in all age groups (*p* = 0.94). Female patients (*p* = 0.03) and subjects with higher education level (*p* = 0.03) had significantly better outcome (Table 1). The median survival was 17 and 9 months respectively, for those with stages I/II (n = 212, 29.5%) and III/IV diseases (n = 506, 70.5%).

**Table 1:** Clinical characteristics and survival months of esophageal squamous cell carcinoma patients.

Variables	Number	Survival	Survival	*p*-value
		Mean ± SD	Median (IQR)	
**Overall**	718	21.2±27.8	10 (5, 25)	
**Gender**				
Female	44	37.2±37.0	23 (5, 58)	0.03
Male	674	20.2±26.7	10 (5, 22)	
**Age (years)**				
≤ 50	181	20.7±28.4	10 (5, 22)	0.94
51–60	201	21.2±27.0	10 (5, 26)	
61–70	169	20.8±25.4	10 (5, 28)	
>70	167	22.3±30.3	9 (4, 23)	
**Education levels**				
< High school	392	19.7±26.1	10 (4,22)	0.03
High school	254	21.2±27.8	10 (5, 25)	
> High school	72	29.1±37.9	13.5 (5, 43)	
**AJCC stage**				
Stage I, II	212	32.6±34.6	17 (7, 47)	<0.0001
Stage III, IV	506	16.5±22.7	9 (4, 18)	
**Alcohol use**				
Never	123	30.9±36.6	12 (5, 46)	0.0009
Ever	595	19.2±25.1	10 (5, 21)	
* Age at starting drinking (years)*				
≤ 20	253	16.7±22.4	10 (5, 18)	0.03
>20	342	21.0±26.9	10 (5, 24)	
* Daily average (drinks/day)*				
≤ 3	226	17.69±22.21	9 (4, 23)	0.23
>3	369	20.15±26.74	10 (5, 20)	
**Cigarette smoking**				
Never	95	28.3±35.5	12 (5, 41)	0.03
Ever	623	20.1±26.3	10 (5–23)	
* Age at starting smoking (years)*				
≤ 20	434	20.3±26.5	9 (4, 24)	0.75
>20	189	19.6±25.6	10 (5, 22)	
* Daily average (cigarettes/day)*				
≤ 20	436	20.6±27.0	10 (4, 24.5)	0.53
>20	187	19.1±24.5	10 (5, 21)	
**Areca chewing**				
Never	397	24.0±29.9	10 (5, 30)	0.002
Ever	321	17.8±24.4	10 (4, 18)	
* Age at starting chewing (years)*				
≤ 20	135	±22.0	9 (4, 16)	0.14
>20	186	19.4±26.0	10 (5, 23)	
* Daily average (nuts/day)*				
≤ 20	202	±21.9	10 (5, 18)	0.73
>20	119	18.4±28.4	9 (3, 19)	

IQR: interquartile range; HR: hazard ratio; CI: confidence interval.

Five hundred and ninety-five patients (82.9%) reported habitual alcohol drinking, 623 (86.8%) cigarette smoking, and 321 (44.7%) areca chewing (Table 1). In univariate analyses, all substance users, including alcohol drinkers (*p* = 0.0009), cigarette smokers (*p* = 0.03), and areca chewers (*p* = 0.002), had adverse overall survival than non-users. Among alcohol drinkers, starting before the age of 20 years was associated with a significantly shorter survival (*p* = 0.03). However, neither the starting age of smoking and areca nut chewing nor the daily amount of these three substances was associated with the patients’ outcome (Table 1). After adjusting for age, gender, education levels and clinical stages only, ESCC patients who consumed alcohol (HR = 1.30, 95% CI = 1.01–1.67, *p* = 0.04) and areca quid (HR = 1.23, 95% CI = 1.03–1.47, *p* = 0.02) but not cigarettes (HR = 1.08, 95% CI = 0.82–1.43, *p* = 0.29) were significantly associated with early death.

**Figure 1:**
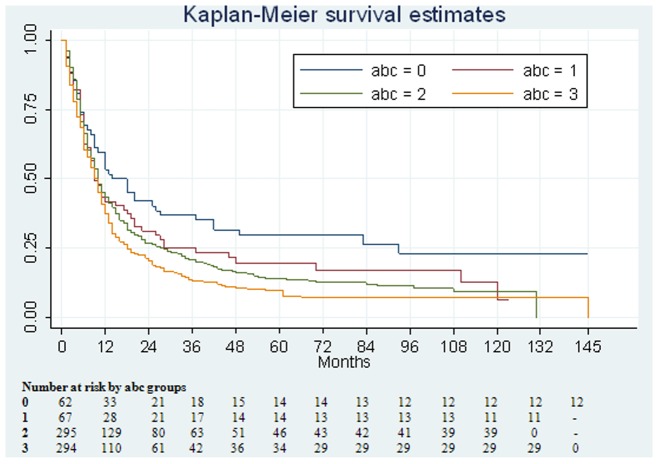
The Kaplan-Meier survival curve categorized by the number of substance use. abc groups: a denotes alcohol drinking; b denotes betel nut chewing; c denotes cigarette smoking.

Table 2 shows survival months of ESCC patients in different combinations of alcohol, tobacco or areca nut. In general, the more the number of substances used, the shorter the survival months. The median survival period was 16 months for non-substance users (n = 62) and 9.5 months for those indulging in all the practices (n = 294). Since the sample size in pure smokers, pure drinkers, and pure chewers were small, we combined them as consuming one substance for the subsequent analyses. The same strategy was applied to the two combined as consumed two substances. Figure 1 shows the Kaplan-Meier survival curves dichotomized by the number of substance uses.

**Table 2:** Survival months of esophageal cancer patients categorized by different combination of alcohol, tobacco or areca nut.

Alcohol	Tobacco	Areca	n	Survival	Survival
				Mean ± SD	Median (IQR)
−	−	−	62	33.5±38.3	16 (5, 49)
−	−	+	2	−	−
−	+	−	43	29.2±35.2	10 (5, 46)
+	−	−	22	16.5±24.2	8 (5, 19)
−	+	+	16	26.7±36.7	9.5 (5.5, 26.5)
+	−	+	9	24.1±37.5	11 (2, 22)
+	+	−	270	21.6±26.7	10 (5, 26)
+	+	+	294	17.1±23.2	9.5 (4, 18)

Compared to non-users, the crude hazard ratios for those using one to three substances were 1.39, 1.50 and 1.80 respectively (*p*-value: 0.01–0.0003) (Table 3). After adjusting for age, gender, education levels and clinical stages, we found that patients who consumed three substances had 1.52-times the risk to die during follow-up than non-users (95% CI = 1.02–2.27; *p = *0.04). Moreover, there was a significant trend toward worse outcome for ESCC patients who used more substances (adjusted *p* for trend = 0.01) (Table 3). As expected, AJCC stage was the most significant predictor for patients’ overall survival (adjusted HR = 1.75, 95% CI = 1.46–2.01; *p*<0.0001). Although the trend remained, neither gender nor education level were significantly associated with survival after adjusting for other covariates (Table 3).

**Table 3:** Hazard ratios of clinical and lifestyle factors for esophageal cancer survival.

Variables	n	Median survival	HR	*p*	Adj. HR*	Adj. *p* * #
		months (IQR)	(95% CI)		(95% CI)	
**Sex**						
Female	44	23 (5, 58)	1.00	0.0007	1.00	0.07
Male	674	10 (5, 22)	1.91		1.52	
			(1.31–2.77)		(0.96–2.41)	
**Education levels**						
< High school	392	10 (4,22)	1.00	−	1.00	−
High school	254	10 (5, 25)	0.91	0.26	0.91	0.33
			(0.77–1.08)		(0.76–1.10)	
> High school	72	13.5 (5, 43)	0.75	0.04	0.87	0.14
			(0.57–0.99)		(0.61–1.08)	
**AJCC stage**						
Stage I, II	212	17 (7, 47)	1.00	<0.0001	1.00	<0.0001
Stage III, IV	506	9 (4, 18)	1.79		1.75	
			(1.50–2.14)		(1.46–2.01)	
**Abc groups**						
0	62	16 (5, 49)	1.00	−	1.00	−
1	67	9 (5, 28)	1.39	0.10	1.17	0.47
			(0.94–2.05)		(0.76–1.80)	
2	295	10 (5, 26)	1.50	0.01	1.25	0.26
			(1.09–2.07)		(0.85–1.83)	
3	294	9.5 (4, 18)	1.80	0.0003	1.52	0.04
			(1.31–2.47)		(1.02–2.27)	

IQR: interquartile range; HR: hazard ratio; CI: confidence interval.

Abc groups: the number of items of alcohol, betel and cigarette consumption.

* Adjusted for age and the other covariates in this table.

# for abc groups, *p* for trend = 0.01.

## Discussion

In this study we found the more substances used, the worse the survival rate. Among them, alcohol drinking seems to be most important, followed by areca nut chewing and smoking. To our knowledge, this is the first report to investigate the influence of areca nut chewing on ESCC survival. Because chewers in Taiwan usually consume alcohol or tobacco and only two patients were pure chewers (Table 2), it was not easy to dissect its independent effect. In addition, there was a high prevalence of smoking (86.8%) and alcohol drinking (82.9%) in our study cohort, but only 43 (6.9%) and 22 (3.7%) of them were pure smokers and drinkers (Table 2). Therefore, their independent effect might be shadowed by the strong influence of clinical stage and over-adjustment for the other two substances in multivariate Cox models. Our previous work has revealed the interactive and multiplicative effect of alcohol, tobacco and areca on the development of ESCC [7], . In this study, we found a significant trend toward worse outcome when one consumed more substances (*p* for trend = 0.01). In Taiwan, areca nut chewers and heavy drinkers usually have lower education level and socioeconomic status. Clinically, they often have less family support when cancer develops and may delay diagnosis or treatment; thus might fare worse. However, we still found that combined substance use was a significant outcome predictor after adjusting for potential confounders, including education level.

Few studies have explored the influence of lifestyle factors on esophageal cancer survival [9]–[11], [17]. A nationwide case-control study in Sweden first reported that previous smokers (HR = 2.1, 95% CI: 1.0–4.4) and low educated subjects (HR = 1.9, 95% CI: 1.1–3.4) had a worse outcome for ESCC. Alcohol drinking and currently smoking were not significant predictors of survival [9]. Another Japanese study supported such findings and further revealed an interaction between heavy smoking and chemoradiotherapy on the prognosis of ESCC [10]. Contrary to these two studies, a recent report of 301 Australian ESCC patients indicated that heavy consumption of alcohol, but not cigarettes, was associated with worse survival [11]. The authors suggested that the inconsistent results might come from limited power of the previous studies because fewer heavy drinkers were present. In that study, current smokers who consumed ≥7 drinks/week had a greatest risk of early death (HR = 3.84, 95% CI = 2.02–7.32), but they could not find a significant additive effect of alcohol and smoking [11]. Although our study had the most ESCC patients so far, and the prevalence of ever-drinkers (82.9%) was similar to the Australian cohort (81.6%), we cannot demonstrate the independent effect of alcohol in multivariate Cox model partially because only 3.4% of them were pure drinkers. Similar to our results, the Australian study showed that gender and education level, which were significant in univariate analysis, were not related to cancer survival after adjusting for covariates [11].

The exact mechanism through which alcohol and areca nut use lead to a worse prognosis remains unclear. One of the possible explanations is that alcohol consumption impairs patient’s nutrient status and immune system, leading to inability to destroy cancer cells. For example, long-term ethanol exposure has significant immunomodulatory effects on the cytotoxic activities of human lymphocytes and decreases the number of peripheral natural killer cells [18], [19]. Moreover, *in vitro* studies have shown that arecoline, one major alkaloid of areca nut, could promote genomic instability through arresting cells at prometaphase with large amounts of misaligned chromosomes and accelerate keratinocyte inflammation by regulating cytokines production such as interleukin-6 and TNF-alpha [20], [21]. Areca nut could not only promote carcinogenesis but also accelerate ESCC migration and lymph node metastasis through activating matrix metalloproteinases-2 and -9 [22]. Further studies are necessary to confirm the direct role of alcohol drinking, areca chewing and possibly tobacco smoking in ESCC survival.

There are several limitations of this study. First, the exposure of interest in our study was measured by questionnaires, and recall bias is likely. The accuracy of information on substance use in this study has been verified by different biomarkers from different specimens to reduce the possibility of information bias [8]. Second, we did not have complete information on cancer treatment, which is a potential confounder of the association between substance uses and overall survival. However, such limitation is not likely to invalidate our findings since there is a standard treatment guideline for esophageal cancer according to clinical staging, which has been adjusted in this study. Finally, because most substance users consumed more than one substance, we were not able to elucidate the independent effects of alcohol, areca nut and cigarettes on ESCC survival.

In conclusion, alcohol drinking plus areca nut chewing and smoking is associated with early death of ESCC. This information is important for clinical oncologists when they manage the ESCC patients with different habits of substance uses. However, such a finding is necessary to be reconfirmed in large-scale prospective cohort studies to recommend a more aggressive treatment for those patients. Future mechanistic studies are also needed to elucidate how these substances lead to a worse outcome.
